# DEVELOPMENT AND TESTING OF AN ACCELEROMETER-BASED POSITIONAL MONITORING SYSTEM

**DOI:** 10.1093/geroni/igz038.1222

**Published:** 2019-11-08

**Authors:** Leighanne Jarvis, Sarah Moninger, Chandra Throckmorton, Juliessa M Pavon, Kevin Caves

**Affiliations:** 1 Duke University, Durham, North Carolina, United States; 2 Duke University Department of Surgery, Durham, North Carolina, United States; 3 Signal Analysis Solutions, LLC, Bahama, North Carolina, United States; 4 Duke University, Durham, United States

## Abstract

Health and fitness are contributing factors to physical resilience, or the ability to resist or recover from functional decline following health stressors. Accelerometer based activity monitors have been used in both the in-patient and outpatient setting to monitor mobility. While using sensors to track mobility is increasing, most clinical settings rely on patient reported outcomes. These measures often under or overestimate movement. The lack of a clinically meaningful way to measure mobility in the in-patient setting is a barrier to improving the mobility of hospitalized individuals. This is especially important when considering that over one-third of hospitalized older adults are discharged with a major new functional disability in performing activities of daily living. Our goal was to automatically determine if the subject is laying, reclining, sitting, standing, and walking to better reflect actual activity. Other platforms and studies indicate the ability to determine a difference in activity vs. inactivity or laying and reclining vs. standing and walking, but not all five phases of movement defined here. The aim of this study was to use accelerometer data to train a machine learning algorithm to automatically classify the postural changes (i.e. laying, reclining, sitting, standing, and walking). Preliminary results demonstrate that our trained algorithm is overall 95% accurate in determining each position from unlabeled data from the subject population. Additionally, this algorithm will be applied to in-patient hospitalized older adults for tracking of positions throughout the day.

